# The Clinicopathologic Importance of Serum Lactic Dehydrogenase in Patients with Gastric Cancer

**DOI:** 10.1155/2014/140913

**Published:** 2014-11-04

**Authors:** Zhi Zhao, Fanghai Han, Shibin Yang, Lixin Hua, Jianhai Wu, Wenhua Zhan

**Affiliations:** Department of Gastrointestinal Surgery, The First Affiliated Hospital of Sun Yat-sen University, Guangzhou 510080, China

## Abstract

*Background*. To explore possible correlation between serum lactate dehydrogenase (SLDH) levels and gastric cancer. *Materials and Methods*. We retrospectively reviewed 365 patients with gastric cancer. The correlation of SLDH levels with clinicopathologic features and survival rate was studied. *Results*. SLDH levels were closely associated with the pathological (p) T stage (*P* = 0.011), metastasis (*P* = 0.012), pTNM stage (*P* = 0.001), and recurrence (*P* = 0.012). Moreover, we found a significant SLDH level difference among Borrmann type (*P* = 0.027), pT stage (*P* = 0.004), lymph node metastasis (*P* = 0.027), metastasis (*P* < 0.001), pTNM stage (*P* = 0.006), and recurrence (*P* = 0.002). In addition, we detected a significant SLDH level difference between alive and dead subgroups (*P* = 0.001). In addition, both univariate analysis and multivariate analysis showed that high SLDH levels were independent prognostic factor. For the subgroup with normal LDH (median point of 157.0 U/L), we detected that the subset with SLDH levels ≥157 U/L (158–245 U/L) showed poorer OS (*P* = 0.005) and DFS (*P* = 0.01) than that of ≤157 subgroup. *Conclusions*. Our results suggest that high SLDH level could be an independent poor prognostic biomarker. Gastric cancer patients with relative high SLDH level (158–245 U/L) were prone to develop a shorter OS and DFS.

## 1. Introduction

Gastric cancer is the fourth most commonly diagnosed malignancy and the second leading cause of cancer death in worldwide [1]. Although the prognosis of gastric cancer has improved in the recent few decades, the overall 5-year survival rate is still poor [2, 3]. The main causes of death in gastric cancer patients are recurrence and metastasis.

Recently, metabolic reprogramming has been recognized as a hallmark of cancer [4–6]. Under aerobic conditions, normal cells seem to generate most of their energy through the mitochondrial oxidative phosphorylation, whereas tumor cells produce a substantial amount of their energy through glycolysis. Cancer cells utilize glycolysis for energy production, even under normoxic conditions, which is known as the “Warburg effect” [7]. This shift of metabolism allows cancer cells to sustain higher proliferative rates. Due to rapid tumor cells divided, high metabolic demands, and tumor avascular area formation, hypoxia is a characteristic property in solid tumors, especially in tumor center [8]. Furthermore, hypoxia further positively facilitated glycolysis [9].

Lactate dehydrogenase (LDH), a cytoplasmic enzyme, reversibly catalyzes the conversion of pyruvate to lactate, which is the last step of glycolysis. Even under normal oxygen concentrations in malignancies, pyruvate transformation to lactate is upregulated [7]. The tumor microenvironment acidification can promote tumor progression and metastasis [10]. There are many tissues in which LDH is widely expressed, such as heart, muscle, and various tumors, and it is detectable in serum. High serum lactate dehydrogenase (SLDH) levels have been reported as a poor prognostic indicator in non-small-cell lung cancer, malignant lymphoma, pancreatic carcinoma, and colorectal cancer [11–14]. Furthermore, current European and American Joint Committee on Cancer (AJCC) recommend SLDH as a staging and progression marker in melanoma [15]. In addition, high LDH protein expression also correlates with poor outcome and metastasis in many solid tumors [16–18].

However, little is known about the association of SLDH levels with gastric cancer. In the present study, we attempted to reveal possible relations between SLDH levels and gastric cancer.

## 2. Materials and Methods

We retrospectively reviewed the medical records of patients who were diagnosed with gastric cancer by the Pathology Department at the First Affiliated Hospital of Sun Yat-sen University between January 2003 and December 2008. Patients were included in the study if they had a pathological diagnosis of gastric cancer, if they had no previous malignancy or second primary tumor, except for early fatal cases, and if they had not received any treatment before they underwent a complete resection of the primary tumor. Patients were excluded from the study if they had a medical history of the same or another type of cancer previously, if they had any of the other conditions in which SLDH levels are increased, or if hemolysis was detected in their blood specimens.

SLDH levels were determined before surgical resection using a Hitachi Automatic Analyzer 7600-020 (Hitachi High-Technologies, Tokyo, Japan) with standard biochemical assays that were recommended by International Federation of Clinical Chemistry and Laboratory Medicine (IFCC). The normal value range of the assay was 120–240 U/L and the coefficient of variance in LDH measurement was <5.0%. A high SLDH value was defined as being above 245 U/L. The 7th TNM classification by the American Joint Committee on Cancer was used for pathologic staging.

All patients had a followup once a month during the first half year and every 3–6 months thereafter; median follow-up period was 35 (range 2 to 87) months for all patients. Overall survival (OS) was defined as the period between the time of surgery and death or was censored at the last followup. Disease-free survival (DFS) was defined as the time from diagnosis to the date of the first disease progression or the date of death or last followup.

The study protocol was approved by the Ethics and Scientific Committee of the First Affiliated Hospital of Sun Yat-sen University and conforms to the Declaration of Helsinki. All patients and their families provided informed written consent for their information to be stored in the hospital database and used for research before surgery.

### 2.1. Statistical Analysis

All statistical calculations were carried out using SPSS 17.0 statistical software. The association between serum LDH levels and various clinicopathologic features was analyzed using the chi-square test. The continuous variables were compared using the Student's *t*-test and one-way ANOVA. The Kaplan-Meier test was employed to evaluate survival rate and the survival rate curve was compared by the log-rank test. A Cox proportional hazard model was applied for multivariate survival analysis. All *P* values <0.05 were considered statistically significant.

## 3. Results

Three hundred sixty-five patients with gastric cancer were finally included in the present study; 240 were males (65.8%) and 125 (34.2%) were females. The median age was 55 years (range 26–77 years). The median follow-up period was 35 (range 6 to 94) months. The baseline clinicopathological characteristics of patients and their correlations with SLDH levels are provided in Tables 1 and 2. In the entire cohort, the SLDH levels were ranged from 81.0 to 295.0 U/L. The mean and median SLDH level were 164 and 157 U/L, respectively. High SLDH levels (>245.0 U/L) were found in 23 patients (16.8%), and 342 patients (83.2%) had SLDH levels within the normal limits.

As shown in Table 1, SLDH levels were closely associated with the pathological (p) T stage (*P* = 0.011), metastasis (*P* = 0.012), pTNM stage (*P* = 0.001), and recurrence (*P* = 0.012). However, no significant correlations were observed between SLDH levels and age, gender, tumor size, Borrmann type, histological type, or pN stage.

Moreover, we found a significant difference in the mean ± standard deviation (SD) SLDH levels among Borrmann type (*P* = 0.027), pT stage (*P* = 0.004), lymph node metastasis (*P* = 0.027), metastasis (*P* < 0.001), pTNM stage (*P* = 0.006), or recurrence (*P* = 0.002) (Table 2). However, there was no significant SLDH level difference among age, gender, tumor size, or histological type.

We further stratified the cohort patients according to their status of followup. Our results showed that there was a significant SLDH level difference between alive and dead subgroups (*P* = 0.001). Moreover, we also detected that patients with metastasis had high SLDH level than those without metastasis (without versus with metastasis, 159.6 ± 34.3 U/L versus 183.1 ± 42.1 U/L, *P* < 0.001).

### 3.1. Survival Analysis

**Figure 1:**
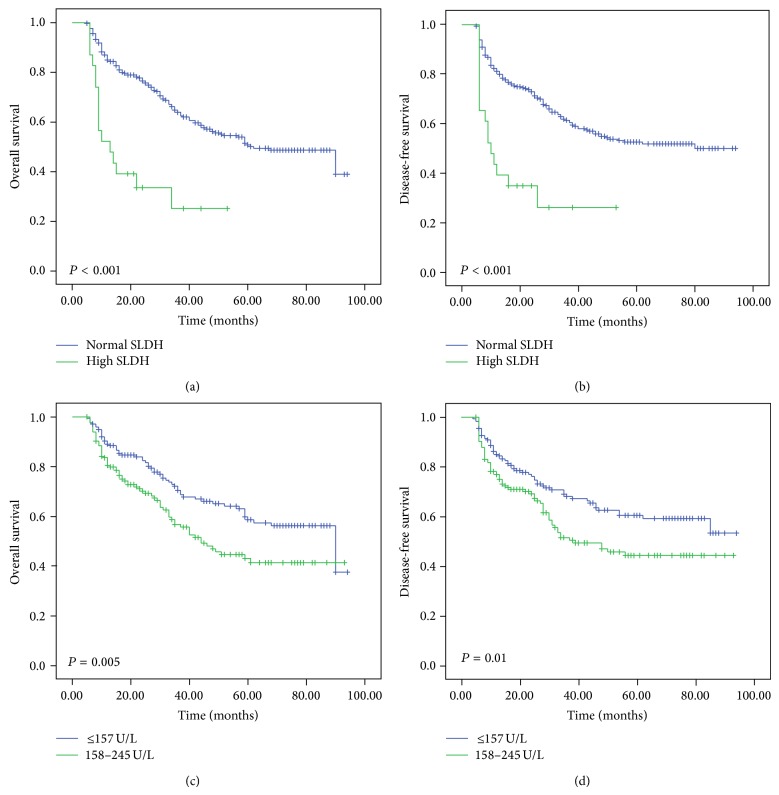
Kaplan-Meier analysis shows that patients with high SLDH have significantly poor overall survival (a) and disease-free survival (b) than those with normal SLDH (*P* < 0.001 for both). Also, patients with SLDH levels ≥157 U/L (158–245 U/L) showed poorer OS (*P* = 0.005) and DFS (*P* = 0.01) than those of ≤157 patients. SLDH: serum lactate dehydrogenase.

Patients with high SLDH levels displayed a significantly shorter overall survival (OS) and disease-free survival (DFS) (*P* < 0.001 for both) compared with patients in the normal SLDH group (Figure 1). Univariate analyses identified SLDH, pT, pN, pM, and pTNM stage as possible prognostic indicators of poor OS and DFS. Multivariate analysis revealed that SLDH was a significant independent prognostic factor for OS (*P* = 0.001) and DFS (*P* < 0.001). Furthermore, pTNM and pM were also identified as an independent prognostic factor for OS and DFS (Table 3).

We further divided the normal SLDH patients (120.0–245.0 U/L) into two subgroups by the median point of 157.0 U/L. We found that the subset with SLDH levels ≥157 U/L (158–245 U/L) showed poorer OS (*P* = 0.005) and DFS (*P* = 0.01) than that of ≤157 subgroup (Figure 1).

## 4. Discussion

In this study, we explored possible associations between SLDH levels and stage and prognosis of human gastric cancer. Our results showed that increased SLDH levels were closely associated with pT, pM, pTNM, recurrence, and poor prognosis. Moreover, we also found that there was significant SLDH level difference among pT, pN, pM, pTNM, and recurrence. When we divided the normal SLDH patients into two subgroups by the median point of the cohort patients' SLDH levels (157.0 U/L), we found that the subgroup with a normal but relative high LDH level (158.0–245.0 U/L) had a poorer OS and DFS than the subset with relatively low SLDH levels (≤157.0 U/L). Furthermore, we detected a significant SLDH level difference between alive and dead subgroups (*P* = 0.001) according to their status of the last followup.

Over the last century, the metabolisms of tumor cells have been studied. Tumor cells have a significant different metabolism from that of the normal tissues, which allowed them to sustain higher proliferation through acquired large quantities of proteins, lipids, nucleotides, and glucose [10]. The mechanisms of metabolic alterations include altered expression, mutation, and posttranslational activation of an enzyme. In tumor cells, LDH is translationally upregulated by hypoxia inducible factor (HIF) as well as myc and is thus regulated by the phosphatidylinositol 3-kinase (PI3K)/serine/threonine kinases AKT/mammalian target of rapamycin (mTOR)/HIF pathway or myc overexpression [9, 19–21]. HIF activation upregulates LDH activity and, in turn, high lactate acid concentrations further positively promote the activation of HIF, suggesting a positive feedback loop between HIF and LDH [22]. Furthermore, HIF overexpression further stimulates VEGF activation [9, 23]. Therefore, high SLDH levels may reflect an upregulated HIF-molecular pathway, more aggressive angiogenesis, and tumor burden, which ultimately lead to poor prognosis in malignant tumors. Mekenkamp et al. found that patients with synchronous metastases from colorectal cancer more often had increased SLDH levels than patients without metachronous metastases, and patients with increased SLDH levels had shorter OS than patients with normal SLDH levels [24]. Shinohara et al. found that increased SLDH levels indicated poor survival in Asian patients with previously untreated metastatic renal cell carcinoma [25]. Danner et al. found that high SLDH level had poorer prognosis in patients with adenocarcinoma or squamous cell lung cancer [11]. Moreover, Scartozzi et al. reported that pretreatment SLDH levels can be used as a predictor of efficacy of first-line bevacizumab-based therapy in metastatic colorectal cancer patients [26]. In our study, we also found that SLDH level was strongly associated with poor OS and DFS. These findings are consistent with those of a previous report. Furthermore, multivariate analysis indicated that SLDH level was an independent prognostic factor for both DFS and OS. Therefore, our findings suggest that SLDH level could be used as a potent prognostic factor for gastric cancer patients. However, Kostakis et al. reported that they did not find any associations between SLDH levels and various clinicopathological parameters of gastric cancer [27]. The incongruent results may have the following reasons. We noticed that, in Kostakis et al.'s study, the sample size was only 40 patients, and a proportion of 47.5% patients were in stages I and II. However, in our study, only 99 (27.1%) patients were in stages I and II. Thus, we inferred that relative small sample size and high ratio of early stage patients may be the reasons accounting for the difference from us.

In accordance with previous studies, our results also showed that increased SLDH levels were significantly associated with early tumor recurrence. Moreover, SLDH levels in recurrence subgroup were significantly higher than subgroup without recurrence. This finding highlights the role of SLDH in predicting disease recurrence in gastric patients. Therefore, it may be useful to monitor SLDH levels in predicting recurrence in the followup of the disease.

In the present study, we found that increased SLDH levels were closely associated with pT, pM, pTNM stage, and recurrence (Table 1). Moreover, we also found that there was significant SLDH level difference among pT, pN, pM, pTNM stage, and survival status (Table 2). A relative high SLDH level indicated an advantaged stage and may reflect heavier tumor burden, more rapid cancer cell proliferation, and higher metabolic demands. Therefore, SLDH levels determinations may provide useful information to patients who have high SLDH level when choosing a postoperative therapy.

Interestingly, in our study, we found that the gastric cancer patients with normal but relative high SLDH levels (158.0–245.0 U/L) had a poor OS and DFS than patients with relative low SLDH levels (≤157.0 U/L). As high SLDH levels may reflect HIF upregulated and tumor burden, a normal but relatively high SLDH level may represent a partial hypoxic condition and relative bulky tumor burden, which was easy to develop a poor OS and DFS.

In conclusion, our results suggest that high SLDH level could be an independent poor prognostic biomarker for gastric cancer patients. Moreover, even for the patient with normal SLDH level, the subgroup with relative high SLDH level (158–245 U/L) was prone to develop a shorter OS and DFS.

## Figures and Tables

**Table 1:** Clinicopathological correlations of SLDH in gastric cancer.

Variable	Normal SLDH (342) *n* (%)	High SLDH (23) *n* (%)	*P*
Age (y)			0.675
<60	208 (93.3)	15 (6.7)	
≥60	134 (94.4)	8 (5.6)	
Gender			0.610
Male	226 (94.2)	14 (5.8)	
Female	116 (92.8)	9 (7.2)	
Tumor size (cm)			0.252
≤4	181 (92.3)	15 (7.7)	
>4	161 (95.3)	8 (4.7)	
Borrmann type			0.092
I	9 (81.8)	2 (18.2)	
II	68 (97.1)	2 (2.9)	
III	226 (94.2)	14 (5.8)	
IV	39 (88.6)	5 (11.4)	
Histological type			0.521
Well diff. ade.	6 (85.7)	1 (14.3)	
Moderately diff. ade.	80 (96.4)	3 (3.6)	
Poorly diff. ade.	249 (92.9)	19 (7.1)	
Undifferentiated ade.	4 (100)	0 (0)	
Squamous cell ca.	3 (100)	0 (0)	
pT stage			0.011^*^
T1	31 (96.9)	1 (3.1)	
T2	29 (100)	0 (0)	
T3	222 (95.3)	11 (4.7)	
T4	60 (84.5)	11 (15.5)	
pN stage			0.106
N0	82 (97.6)	2 (2.4)	
N1	148 (94.3)	9 (5.7)	
N2	72 (92.3)	6 (7.7)	
N3	40 (97.0)	6 (1.3)	
Metastasis			0.012^*^
No	279 (95.5)	13 (4.5)	
Yes	63 (86.3)	10 (13.7)	
pTNM stage			0.001^*^
I	41 (97.6)	1 (2.4)	
II	54 (94.7)	3 (5.3)	
III	155 (97.5)	4 (2.5)	
IV	92 (86.0)	15 (14.0)	
Recurrence			0.012^*^
No	210 (96.3)	8 (3.7)	
Yes	132 (89.8)	15 (10.2)	

SLDH: serum lactate dehydrogenase; ade.: adenocarcinoma; diff.: differentiated; ca.: carcinoma; ^*^ *P* < 0.05.

**Table 2:** The SLDH levels in gastric cancer patients.

Variable	*n* (%)	SLDH (U/L, mean ± SD)	*P*
Age (y)			0.675^a^
<60	223 (61.1)	163.8 ± 36.5	
≥60	142 (38.9)	165.0 ± 38.2	
Gender			0.334^a^
Male	240 (94.2)	162.9 ± 37.2	
Female	125 (92.8)	166.9 ± 37.1	
Tumor size (cm)			0.214^a^
≤4	196 (53.7)	154.6 ± 38.2	
>4	169 (46.3)	159.3 ± 31.7	
Borrmann type			0.027^*^^b^
I	11 (3.0)	184.5 ± 42.0	
II	70 (19.2)	155.0 ± 32.5	
III	240 (65.8)	164.8 ± 37.3	
IV	44 (12.1)	171.0 ± 39.5	
Histological type			0.242^a^
Well diff. ade.	7 (1.9)	154.2 ± 45.9	
Moderately diff. ade.	83 (22.7)	162.2 ± 33.1	
Poorly diff. ade.	268 (73.4)	165.6 ± 38.0	
Undifferentiated ade.	4 (1.1)	171.0 ± 43.2	
Squamous cell ca.	3 (0.8)	120.0 ± 6.55	
pT stage			0.004^*^^a^
T1 + T2	61 (16.7)	151.9 ± 32.4	
T3 + T4	304 (83.3)	166.8 ± 37.6	
Lymph node metastasis			0.027^*^^a^
No	84 (23.0)	156.4 ± 31.9	
Yes	281 (43.0)	166.6 ± 36.3	
Metastasis			<0.001^*^^a^
No	292 (80.0)	159.6 ± 34.3	
Yes	73 (20.0)	183.1 ± 42.1	
pTNM stage			0.006^*^^a^
I + II	99 (16.7)	155.5 ± 34.1	
III + IV	266 (83.3)	167.6 ± 37.7	
Recurrence			0.002^*^^a^
No	218 (59.7)	159.1 ± 34.0	
Yes	147 (40.3)	172.0 ± 40.3	
Survival			0.001^*^^a^
Alive	216 (59.2)	158.6 ± 33.5	
Dead	149 (40.8)	172.5 ± 40.5	

SLDH: serum lactate dehydrogenase; ade.: adenocarcinoma; ca.: carcinoma; SD: standard deviation.

^a^Independent *t*-test; ^b^one-way ANOVA test; p: pathological; ^*^ *P* < 0.05.

**Table 3:** Univariate and multivariate analyses of prognostic variables in gastric cancer patients.

Parameters	Univariate analysis	Multivariate analysis	
	*P*	HR (95% CI)	*P*
OS			
Age (≥60)	0.676	—	0.800
Gender	0.076	—	0.226
SLDH	<0.001^*^	2.476 (1.450–4.228)	0.001^*^
Tumor size	0.112	—	0.105
Borrmann type	0.329	—	0.070
Histological type	0.561	—	0.521
pT stage	<0.001^*^	—	0.322
pN stage	0.004^*^	—	0.630
pM stage	<0.001^*^	2.157 (1.454–3.201)	<0.001^*^
pTNM	<0.001^*^	1.920 (1.548–2.381)	<0.001^*^
DFS			
Age (≥60)	0.680	—	0.938
Gender	0.097	—	0.250
SLDH	<0.001^*^	2.209 (1.292–3.778)	0.004^*^
Tumor size	0.785	—	0.136
Borrmann type	0.381	—	0.061
Histological type	0.632	—	0.475
pT stage	<0.001^*^	—	0.197
pN stage	0.002^*^	—	0.455
pM stage	<0.001^*^	1.926 (1.306–2.840)	0.001^*^
pTNM	<0.001^*^	1.880 (1.516–2.330)	<0.001^*^

CI: confidence interval; HR: hazard ratio; OS: overall survival; DFS: disease-free survival; p: pathological.

SLDH: serum lactate dehydrogenase. ^*^ *P* < 0.05.
